# 475. Boosting ID Fellow Confidence in Outpatient Travel Medicine and Latent Tuberculosis Clinic Encounters through Low-Stakes, High-Fidelity Role Play

**DOI:** 10.1093/ofid/ofaf695.163

**Published:** 2026-01-11

**Authors:** David A Lindholm, Joseph Yabes, Mary B Ford, Jamie L Geringer, John Kiley

**Affiliations:** Uniformed Services University of the Health Sciences, San Antonio, TX; Brooke Army Medical Center, San Antonio, Texas; Brooke Army Medical Center, San Antonio, Texas; Uniformed Services University of the Health Sciences F. Edward Hebert School of Medicine, San Antonio, Texas; BAMC, San Antonio, Texas

## Abstract

**Background:**

Outpatient clinical encounters for pre-travel counseling and the evaluation of latent tuberculosis infection (LTBI) are common in infectious disease practice but not in general internal medicine practice. As such, incoming infectious disease fellows may lack confidence in their approach to these unique encounters. To address this, we implemented a low-stakes role play into our introductory ID fellowship curriculum, using Kern’s model of curriculum development and a modified Sawyer’s pedagogical framework.

**Figure:**
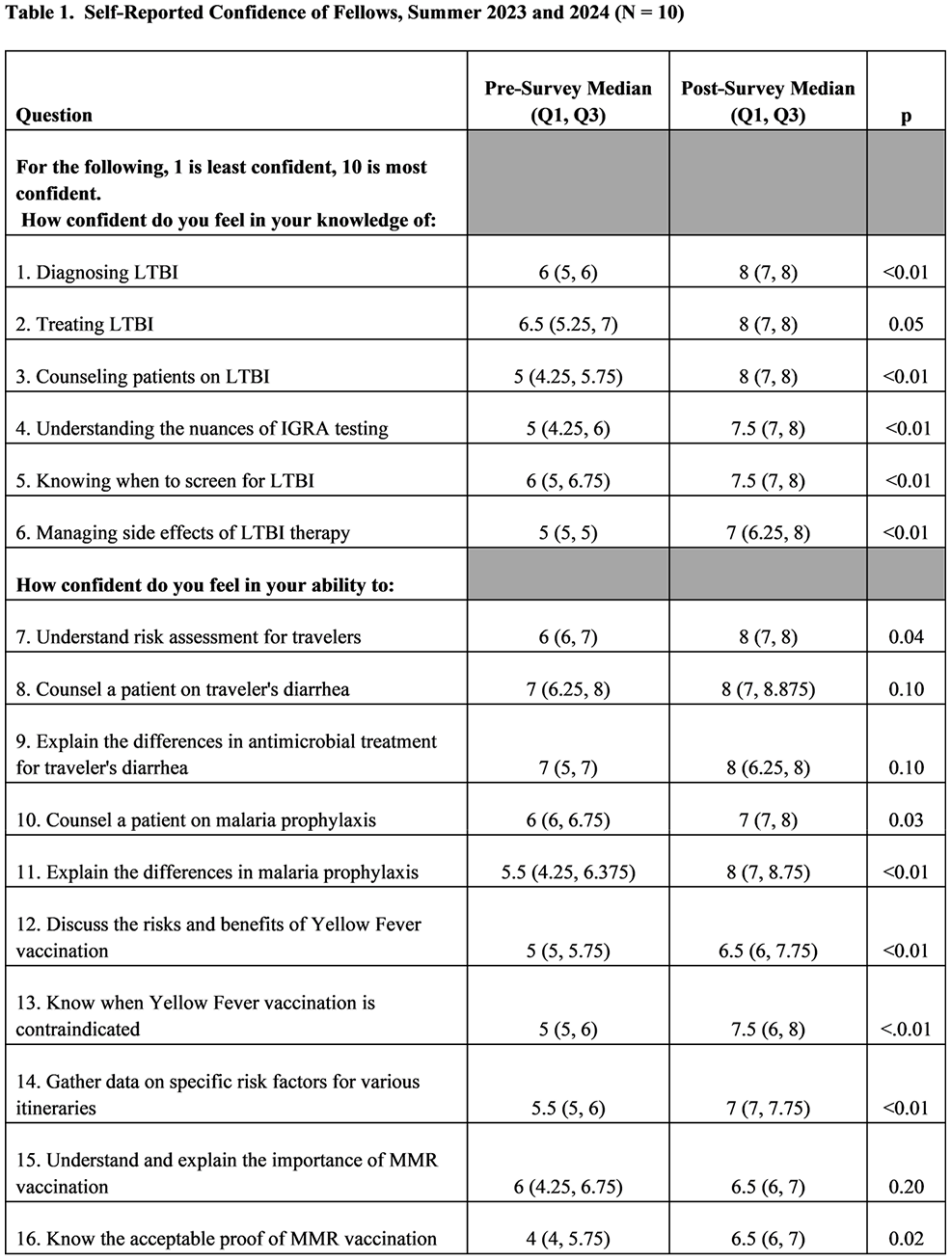
Abbreviations: IGRA, interferon-gamma release assay; LTBI, latent tuberculosis infection; MMR, measles, mumps, and rubella

**Methods:**

In the first month of fellowship, a core lecture series on pre-travel counseling and LTBI preceded low-stakes, formative role-play sessions, which paired one faculty (the “patient”) with one fellow (the “provider”) to simulate discrete, standardized clinical scenarios for each topic. Real-time and/or post-encounter feedback was provided, as needed. Self-reported fellow confidence in knowledge and abilities were surveyed on a 10-point Likert scale pre- and post-session. Data were analyzed using the Mann-Whitney U test.

**Results:**

10 fellows (5/first-year class in each of the 2023-2024 and 2024-2025 academic years) participated. Fellow confidence in their self-assessed knowledge increased significantly in all 6 measured domains (Table 1). Fellow confidence in their self-assessed ability increased significantly in 7/10 domains. Confidence did not differ in their perceived ability to counsel a patient on traveler’s diarrhea, explain the difference in treatment options for traveler’s diarrhea, or to understand and explain the importance of the measles, mumps, and rubella (MMR) vaccination. Free-text feedback showed that fellows valued the formative and realistic nature of the encounters, with recommendations for allowing observation of a clinical encounter by an experienced physician and access to additional preparatory materials.

**Conclusion:**

This low-stakes, high-fidelity role-play innovation demonstrated improved fellow confidence in their knowledge and abilities to approach common outpatient ID scenarios, and it was well-received by the fellows. Incorporating role-play clinical scenarios to fill curriculum gaps may benefit other programs as a tool to build clinical skills and guide further curriculum development.

**Disclosures:**

All Authors: No reported disclosures

